# Sperm DNA damage compromises embryo development, but not oocyte fertilisation in pigs

**DOI:** 10.1186/s40659-022-00386-2

**Published:** 2022-04-01

**Authors:** Yentel Mateo-Otero, Marc Llavanera, Sandra Recuero, Ariadna Delgado-Bermúdez, Isabel Barranco, Jordi Ribas-Maynou, Marc Yeste

**Affiliations:** 1 Biotechnology of Animal and Human Reproduction (TechnoSperm), Institute of Food and Agricultural Technology, University of Girona, S17003 Girona, Spain; 2 Unit of Cell Biology, Department of Biology, Faculty of Sciences, University of Girona, S17003 Girona, Spain; 3 Department of Veterinary Medical Sciences, Via Tolara di Sopra 50, Ozzano dell’Emilia, T40064 Bologna, Italy; 4 Catalan Institution for Research and Advanced Studies (ICREA), S08010 Barcelona, Spain

**Keywords:** Sperm DNA damage, Embryo development, Oocyte fertilisation, Porcine

## Abstract

**Background:**

The assessment of sperm DNA integrity has been proposed as a complementary test to conventional mammalian semen analysis. In this sense, single-strand (SSB) and double-strand (DSB) DNA breaks, the two types of sperm DNA fragmentation (SDF), have been reported to have different aetiologies and to be associated to different fertility outcomes in bovine and humans. Considering that no studies in porcine have addressed how SDF may affect sperm quality and fertility outcomes, the present work aimed to determine the impact of global DNA damage, SSB and DSB on sperm quality and in vitro fertilising ability. To this end, 24 ejaculates (one per boar) were split into three aliquots: the first was used to assess sperm quality parameters through a computer-assisted sperm analysis (CASA) system and flow cytometry; the second was used to perform in vitro fertilisation, and the third, to evaluate sperm DNA integrity using alkaline and neutral Comet assays.

**Results:**

The results showed that global DNA damage negatively correlates (*P* < 0.05) with normal sperm morphology (R = − 0.460) and progressive motility (R = − 0.419), and positively with the percentage of non-viable sperm (R = 0.507). Multiple regression analyses showed that non-viable sperm were related to SSB (β = − 0.754). In addition, while fertilisation did not seem to be affected by sperm DNA integrity, global DNA damage, DSB and SSB were found to be correlated to embryo development outcomes. Specifically, whereas global DNA damage and DSB negatively affected (*P* < 0.05) the later preimplantation embryo stages (percentage of early blastocyst/blastocyst D6: for global DNA damage, R = − 0.458, and for DSB, R = − 0.551; and percentage of hatching/hatched blastocyst D6: for global DNA damage, R = − 0.505, and for DSB, R = − 0.447), global DNA damage and SSB had a negative impact (*P* < 0.05) on the developmental competency of fertilised embryos (R = − 0.532 and R = − 0.515, respectively). Remarkably, multiple regression analyses supported the associations found in correlation analyses. Finally, the present work also found that the inclusion of Comet assays to the conventional sperm quality tests improves the prediction of blastocyst formation (AUC = 0.9021, *P* < 0.05), but not fertilisation rates (*P* > 0.05).

**Conclusion:**

Considering all these findings, this work sets a useful model to study how SDF negatively influences fertility.

## Background

Over the last decades, research on the improvement of assisted reproductive techniques (ART) has gained much relevance due to the decreased human fertility rates and the improvement of profitability in livestock reproduction [1–3]. In this realm, infertility has been typically considered as a multifactorial pathological condition involving the combined effect of male and female factors in equal parts [4]. Focusing on the male factor, mounting evidence indicates that the assessment of conventional sperm quality parameters does not efficiently predict the efficiency of ART [5, 6]. For this reason, more complex tests, including the evaluation of sperm functionality by flow cytometry [7] or the assessment of sperm DNA integrity [8], have been developed. Despite that, controversial results reported by some clinical studies have led scientific societies to pronounce different opinions about the suitability of including these advanced tests into the human semen routine analysis [9–13].

Sperm DNA fragmentation (SDF) is a genotoxic insult occurring in response to intrinsic or extrinsic oxidative stress, as a result of chromatin remodelling during spermiogenesis or due to enzymatic activity and apoptotic-like processes [14, 15]. Recently, the use of advanced methods that allow discriminating different types of sperm DNA damage has shown that single-strand (SSB) and double-strand (DSB) DNA breaks may have different aetiologies and may lead to reproductive consequences [16]. On the one hand, SSB are an oxidative-related DNA damage mainly caused by oxidative stress, which is produced by the imbalance between reactive oxygen species (ROS) and antioxidants. The ROS are highly-reactive small radicals that interact with nitrogenized bases of the DNA, forming DNA adducts such as 8-hydroxy-2′deoxyguanosine (8OHdG), which are excised and generate a SSB [17]. This effector mechanism usually leads to an extensive DNA damage distributed alongside the sperm genome, both in toroidal and toroid linker regions, resulting in lack of pregnancy or an increase of conception time [16, 18]. On the other hand, DSB have been shown to be highly localised at the toroid linker regions and is probably triggered by the enzymatic activity occurring at meiotic or post-meiotic stages. Remarkably, DSB has been reported to increase the risk of implantation failure and miscarriage, and is associated to low embryo quality [18–22].

To date, many methods with different molecular basis have been developed to evaluate sperm DNA fragmentation, the most used ones being (1) the terminal deoxynucleotidyl transferase dUTP nick end labelling (TUNEL), (2) the sperm chromatin structure assay (SCSA), (3) the sperm chromatin dispersion (SCD) and (4) the Comet assay. Despite the high standardization of TUNEL, SCSA and SCD tests, their major drawback is their inability to separately evaluate SSB/DSB [16, 21]. Contrarily, while the Comet assay is a less standardized method with wide variations between laboratories, it can be performed under alkaline or neutral pH to specifically discriminate between SSB and DSB [16, 21]. Despite the high amount of studies conducted in mammalian species using different SDF methods, their different molecular basis and the lack of consensus regarding the cut-off values have led to controversial conclusions about their usefulness in ART. In effect, while some authors find a negative relationship between DNA fragmentation and fertility [23, 24], others do not observe such an association [25, 26]. Yet, a recent meta-analysis conducted in a substantially high number of human patients showed that these discrepancies may not only reside in the method of analysis of DNA fragmentation, but could also be explained by the different association between SDF and in vitro fertilisation (IVF) or intracytoplasmic sperm injection (ICSI) [27]. Thus, while there is a consensus on the detrimental impact of SDF on natural pregnancies and IVF outcomes, this is not the case of ICSI [27]. This difference is likely to be explained by the technical differences between ICSI and IVF, as the former involves the selection of a single spermatozoon based on its motility and morphology, traits that have been shown to be negatively correlated to DNA damage [28, 29]. To bring light into the topic, a recent systematic review pointed out that oxidative DNA damage induced in sperm from different mammalian species has an adverse effect on IVF and ICSI embryos [30]. Similarly, inconsistent data about the impact of SDF on sperm quality have been reported. Indeed, whereas some observed close associations between SDF and seminogram parameters [19, 28, 31–37], others did not [38, 39].

Besides studies conducted in humans, the impact of SSB and DSB on fertility outcomes has been scarcely evaluated in other mammalian species. Establishing the effects and the potential relationship between the different types of DNA damage and sperm quality parameters, fertilisation and even embryo development could, however, open the possibility of using animal models to evaluate the precise genotoxic DNA damage induced by extrinsic factors, their effector mechanism and their impact on fertility rates [40]. In this sense, porcine species has been previously proposed as a suitable animal model for the study of sperm capacitation, fertilisation and male infertility [41]. While a recent work carried out by our research group characterised the two types of DNA breaks in pig sperm [42], no study has explored their potential relationship to IVF outcomes. The aim of the present study, therefore, was to determine the effects of SSB and DSB on: (i) sperm quality parameters; (ii) oocyte fertilisation; and (iii) embryo development.

## Results

### Relationship of global DNA damage, SSB and DSB with sperm quality parameters

**Fig. 1:**
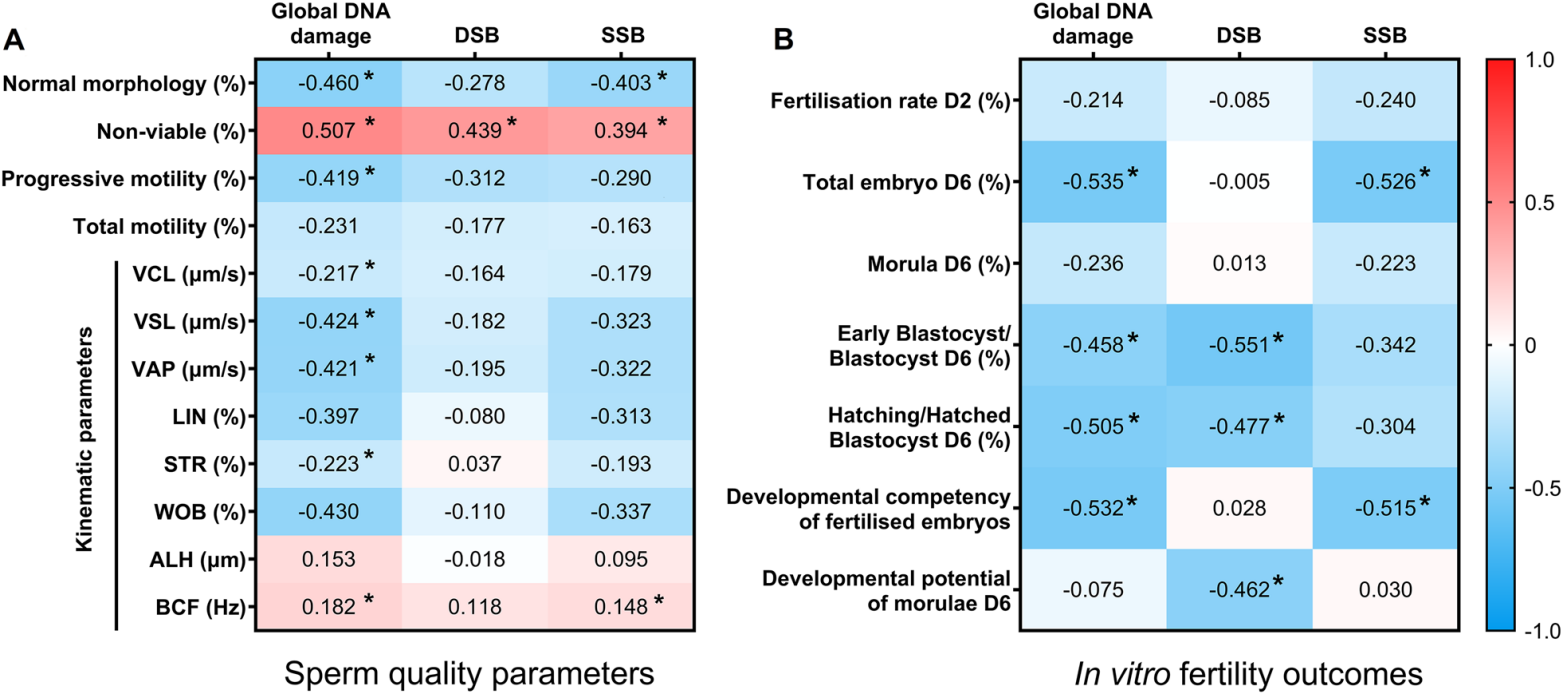
**A** Correlation heatmap of sperm quality parameters (including sperm morphology, motility and viability) and global DNA damage, double- (DSB) and single-SDF (SSB). **B** Correlation heatmap of in vitro fertility outcomes evaluated on day 2 (D2) and Day 6 (D6) and global DNA damage, DSB and SSB. Semen samples of 24 AI-boars split in three aliquots: the first was used to assess sperm quality after semen samples arrived at the laboratory, the second was used to perform in vitro fertility procedures, and the third was used to evaluate alkaline and neutral Comet. The colour saturation of red to blue represents the Pearson correlation coefficients (R) from 1 to − 1, respectively. Significant correlations (*P* < 0.05) are marked with *

The first aim of the present study was to evaluate the potential relationship between SDF and sperm quality parameters, in terms of sperm morphology, motility and viability. To this end, the global DNA damage was calculated as Olive Tail moment (OTM) from the alkaline Comet, DSB were evaluated using the OTM from neutral Comet and, finally, SSB were calculated by subtracting the neutral Comet OTM from the alkaline Comet OTM. Next, Spearman correlations were calculated with each of these parameters (Fig. 1A). Moreover, because a strong correlation between global DNA damage and SSB was observed (R = 0.925; *P* = 0.925), multiple regression analyses including SSB, DSB and morphology, motility or viability variables were conducted.

Regarding sperm morphology, positive correlations (*P* < 0.05) between the percentage of sperm with abnormal morphology and global DNA damage and SSB were observed (R = 0.460 and R = 0.403, respectively). Regarding sperm motility, only global DNA damage was found to exhibit a negative correlation (*P* < 0.05) with the percentage of sperm with progressive motility (R = − 0.419) and specific sperm motility kinematic parameters, including straight-line velocity (VSL), average path velocity (VAP), percentage of linearity (LIN) and motility parameter wobble (WOB) (R = − 0.424, R = − 0.421, R = − 0.397, R = − 0.430, respectively). Finally, the percentage of non-viable sperm was positively correlated (*P* < 0.05) with global DNA damage, DSB and SSB (R = 0.507, R = 0.439 and R = 0.394, respectively). Multiple regression analyses showed no association between SSB or DSB and morphology or motility (*P* > 0.05), but did find an association between SSB and sperm viability (β = − 0.754; *P* = 0.019).

### Relationship of global DNA damage, SSB and DSB with IVF outcomes

This study also explored the effect of SDF on oocyte fertilisation and embryo development. To this end, Spearman correlations of global DNA damage, SSB and DSB with IVF outcomes were calculated (Fig. 1B).

First, no correlation (*P* > 0.05) between fertilisation rate on day 2 and any of the SDF indices evaluated was found. On the contrary, several correlations between embryo development and the different SDF types were observed. Specifically, the total number of embryos on day 6 was negatively correlated (*P* < 0.05) to both global DNA damage and SSB (R = − 0.535 and R = − 0.526, respectively). In addition, the different SDF types were also observed to have an influence on specific embryo stages. In effect, both global DNA damage and DSB exhibited the same pattern, showing a negative correlation (*P* < 0.05) with the percentages of early blastocysts/blastocysts and hatching/hatched blastocysts (for global DNA damage: R = − 0.468 and R = − 0.505, respectively; for DSB: R = − 0.551 and R = − 0.477, respectively), but not with the percentages of morula (*P* > 0.05). On the other hand, SSB were not found to correlate (*P* > 0.05) with any of the embryo stages on day 6. Considering the correlation found between global and DNA damage and SSB (shown in “Relationship of global DNA damage, SSB and DSB with sperm quality parameters” section), multiple linear regression analysis were subsequently conducted including SSB, DSB, day 2 fertilization rate and day 6 embryo outcomes (total number of embryos, morulae, early blastocysts/blastocysts and hatching/hatched blastocysts). The analysis confirmed that no association between fertilization rate on day 2 and SSB or DSB existed (*P* > 0.05), and showed an association between SSB and total number of embryos on day 6 (β = − 0.141; *P* = 0.010), between DSB and early blastocysts/blastocysts (β = − 0.042; *P* < 0.001), and between DSB and hatching/hatched blastocysts (β = − 0.140; *P* = 0.018).

To evaluate the developmental potential of morulae, the percentage of early blastocysts/blastocysts plus hatched/hatching blastocysts was divided by the percentage of morulae. DSB were negatively correlated with developmental competency (R = − 0.418; *P* = 0.023), but neither global DNA damage nor SSB showed such a relationship (*P* > 0.05). The multiple regression analysis also showed the association of this parameter to DSB (β = − 0.890; *P* = 0.044), but not to SSB (*P* > 0.05), with the developmental potential of morulae.

Finally, the developmental competency of fertilised embryos was calculated as the ratio between the total number of embryos on day 6 and the total number of embryos on day 2. Whereas global DNA damage and SSB were found to negatively correlate (*P* < 0.05) with the embryo developmental rate (R = − 0.532 and R = − 0.515, respectively), DSB did not (*P* > 0.05). The results obtained from the multiple regression analysis were similar (*P* > 0.05 for DSB; β = − 0.065 and *P* = 0.042 for SSB).

### Prediction of in vitro fertility outcomes through conventional sperm quality parameters and Comet

The last aim of this study was to evaluate whether the inclusion of alkaline and neutral Comet tests to the conventional semen analysis (which comprises the assessment of sperm morphology, motility, and viability) improved the prediction of IVF outcomes, specifically, fertilisation rate on day 2 and percentage of total blastocysts on day 6.

**Fig. 2:**
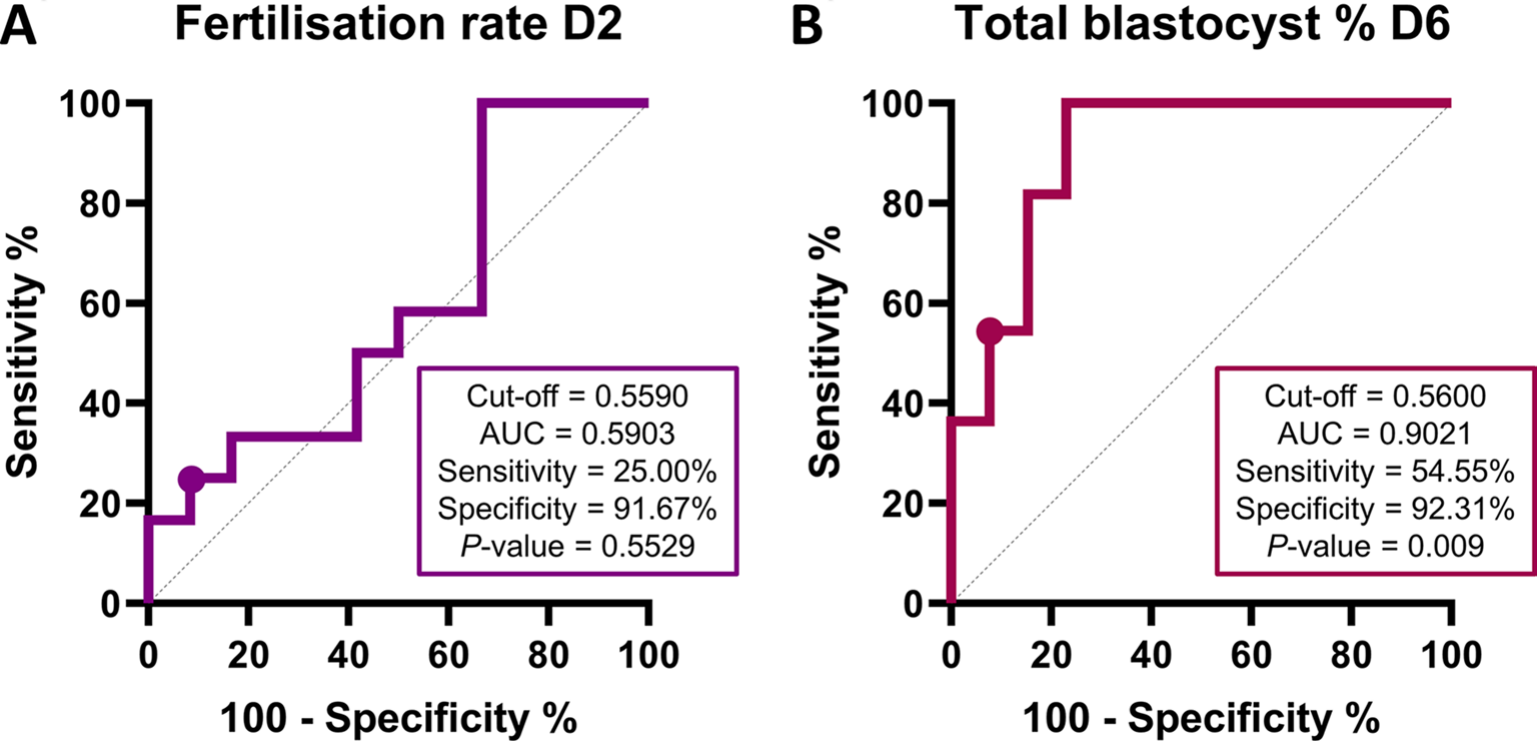
Receiver operating characteristic (ROC) curve analysis for fertilisation rate on day 2 (**A**) and percentage of total blastocyst on day 6 (**B**). They show the ability of conventional sperm quality parameters combined with Alkaline and Neutral Comet assay to discriminate the fertilisation rate and the percentage of total blastocysts on day 6. *AUC* area under the curve

First, semen samples were divided by the median of fertilisation rate on day 2 in two groups: low (ranging 20.0–29.3%, n = 12) and high (ranging 32.5–63.4%, n = 12) fertilisation rate. Then, a Receiver Operating Characteristic (ROC) curve was elaborated for each sperm quality parameter (Table 1). The ROC curve analysis showed that only the percentage of total motility was able to predict the fertilisation rate on day 2 (*P* < 0.05), showing a good discriminant value with an Area Under the Curve (AUC) of 0.8750. In addition, none of the Comet assays exhibited a significant AUC (*P* > 0.05). Following this, principal components were extracted to elaborate a combination of all the parameters (sperm motility, morphology, viability and alkaline and neutral Comet), and ROC curve analysis was redone for the first component. The combination of all parameters, however, showed no significant AUC (Fig. 2A).

**Table 1:** Receiver operating characteristic (ROC) for each sperm quality parameter to predict fertilisation rate on day 2

	AUC (95% CI)	*P* value	Cut-off value (%)	Sensitivity (95% CI)	Specificity (95% CI)	ODDs ratio
Morphology	0.5556 (0.3174–0.7938)	0.6442	94.53	58.33% (31.95–80.67%)	66.67% (39.06–86.19%)	1.750
Total motility	0.8750 (0.7246–1.000)	0.0018	88.61	75.00% (46.77–91.11%)	91.67% (64.61–99.57%)	9.000
Progressive motility	0.5625 (0.3211–0.8039)	0.6033	78.14	41.67% (19.33–68.05%)	91.67% (64.61–99.57%)	5.000
Viability	0.6806 (0.4476–0.9135)	0.1333	90.03	66.67% (39.06–86.19%)	75.00% (46.77–91.11%)	2.667
OTM alkaline-neutral Comet	0.5833 (0.3458–0.8208)	0.4884	10.80	25.00% (8.894–53.23%)	91.67% (64.61–99.57%)	3.000
OTM neutral Comet	0.5486 (0.3081–0.7892)	0.6861	3.658	33.33% (13.81–60.94%)	91.67% (64.61–99.57%)	4.000
Combination (Component 1)	0.5903 (0.3543–0.8261)	0.5529	0.5590	25.00% (8.89–53.35%)	91.67% (64.61–99.57%)	3.000

*AUC* area under the curve; *CI* confidence interval; *OTM* olive tail moment

Next, semen samples were categorized in two groups considering the median of the percentage of total blastocysts on day 6 (calculated as the sum of the percentage of early blastocysts/blastocysts and that of hatched/hatching blastocysts): low (ranging 2.9–10.0%, n = 13) and high (ranging 11.4–29.0%, n = 11). Then, a ROC curve analysis for each sperm quality parameter was run (Table 2). In this case, the percentages of total motile and viable sperm exhibited a good discriminant predictive value with an AUC of 0.8392 and 0.8671, respectively (*P* < 0.05). Moreover, a tendency (*P* = 0.0597) for the AUC of both sperm morphology and progressive motility was found, with an associate AUC of 0.77273 in both cases. Additionally, although only the neutral Comet assay showed a significant AUC (*P* < 0.05), displaying a good discriminant value to predict the percentage of total blastocysts on day 6 with an AUC of 0.8042, a tendency (*P* = 0.0597) for the AUC of the alkaline-neutral OTM was observed (AUC = 0.7273). When principal components were extracted to address whether a combined model of all the parameters predicted the percentage of total blastocysts on day 6, an excellent discriminant value for the first component, with an AUC of 0.9021, was found (*P* < 0.05; Fig. 2B).

**Table 2:** Receiver operating characteristic (ROC) for each sperm quality parameter to predict the total blastocyst percentage on day 6

	AUC (95% CI)	P value	Cut-off value (%)	Sensitivity (95% CI)	Specificity (95% CI)	ODDs ratio
Morphology	0.7273 (0.5077–0.9468)	0.0597	94.18	81.82% (52.30–96.77%)	76.92% (49.74–91.82%)	3.545
Total motility	0.8392 (0.6734–1.000)	0.0050	91.39	45.45% (21.27–71.99%)	92.31% (66.69–99.61%)	5.909
Progressive motility	0.7273 (0.5242–0.9304)	0.0597	80.19	36.36% (15.17–64.62%)	92.31% (66.69–99.61%)	4.727
Viability	0.8671 (0.7251–1.000)	0.0024	91.37	63.64% (35.38–84.83%)	92.31% (66.69–99.61%)	8.273
OTM alkaline—neutral Comet	0.7273 (0.5175–0.9370)	0.0597	11.67	45.45% (21.27–71.99%)	92.31% (66.69–99.61%)	5.909
OTM neutral	0.8042 (0.6024–1.000)	0.0117	2.72	90.91 (62.26–99.53%)	84.62 (57.77–97.27%)	5.909
Combination (Component 1)	0.9021 (0.7773–1.000)	0.0009	0.56	54.55 (28.01–78.73%)	92.31 (66.69–99.61%)	7.091

*AUC* area under the curve; *CI* confidence interval; *OTM* olive tail moment

## Discussion

Sperm DNA fragmentation has been shown to have a great impact on natural fertility outcomes [21, 27] and sperm quality parameters [28] in humans. In porcine, although it has been reported that global DNA damage affects litter size [43, 44], the effects of specific DNA breaks on sperm fertilising ability and embryo development have not been addressed. To this end, the present work aimed to explore the relationship between global DNA damage, SSB and DSB evaluated using the Comet assay and sperm quality parameters and IVF outcomes. The results of the present study indicate that: (i) the incidence of global DNA breaks correlates with sperm quality, assessed in terms of sperm morphology, motility and viability; (ii) SDF is not correlated to the sperm ability to fertilise oocytes; (iii) global DNA damage and DSB may disturb late pre-implantation embryo development, and global DNA damage and SSB have a negative impact on embryo developmental competency from day 2 to day 6; and (iv) the inclusion of Comet assays to the conventional spermiogram parameters improves the prediction of IVF success, specifically blastocyst formation.

There is conflicting evidence about the impact of sperm DNA breaks on sperm quality in humans [28, 31–36, 38, 39]. In porcine, only one study from our group addressed this, finding no correlation between sperm quality and neutral Comet OTM and only a weak correlation between sperm kinematic parameters and Alkaline Comet OTM [45]. Moreover, no study has investigated the relationship of global DNA damage, SSB and DSB with sperm quality in livestock. The present report found a positive correlation between the incidence of SSB and the percentage of morphologically abnormal sperm, which were not confirmed by the multiple regression analysis. Previous studies performed in humans [28, 34–37] and cattle [46] showed increased levels of sperm DNA breaks in semen samples with a high percentage of sperm with morphological abnormalities. Yet, it is worth mentioning that none of the aforementioned studies evaluated the correlation between sperm morphology and SSB or DSB independently. For this reason, the present study is the first suggesting a possible positive relationship between SSB and sperm morphological abnormalities. A direct cause-effect, however, was not observed through the multiple regression analysis, thus suggesting that a third player influencing those alterations should not be discarded. In addition, the present work also assessed the relationship between sperm motility parameters and DNA integrity, finding a negative correlation between the incidence of global DNA breaks, the percentage of sperm with progressive motility and several motility kinematic parameters. These results are in agreement with a previous work in pig sperm, in which log-transformed DNA fragmentation index assessed through SCSA negatively correlated with sperm motility [43]. However, unlike other studies in which SSB, but not localised DSB, negatively influenced progressive motility in humans [19], the results of this study found no relationship between any of the specific DNA break types and motility parameters. The relationship found between global DNA damage and motility in our study is, nevertheless, in accordance with previous reports in humans [31–33]. Again, a lack of cause-effect association between SSB and DSB and sperm motility was observed, suggesting that both parameters could be altered upon exposure to a third causative mechanism, which may be, for instance, oxidative stress [47]. Finally, the current work also identified a negative correlation between the incidence of global DNA breaks, SSB and DSB and the percentage of non-viable sperm. Although, to the best of our knowledge, no previous study addressed whether the specific DNA breaks are related to sperm viability, earlier reports in humans found a strong negative correlation between DNA fragmentation and this sperm parameter [48]. In this case, SSB were found to be associated to the percentage of non-viable sperm in a multiple regression analysis, evidencing that cell death is closely related to DNA damage.

The impact of SDF on fertility has been extensively studied in humans [23, 24, 27] and DNA fragmentation evaluated with SCSA has been reported to be negatively related to farrowing rate and litter size in productive species [43, 49, 50]. Hence, after investigating the link between DNA breaks and conventional spermiogram parameters, we hypothesised that the different types of SDF could also lead to different outcomes after IVF. Our results showed that, while fertilisation rate on day 2 was not caused by or related to sperm DNA damage, global DNA breaks and SSB negatively influenced the number of embryos obtained on day 6. These findings indicate that, while DNA integrity does not affect the sperm ability to fertilise oocytes, it may compromise embryo development, as it has been already posited before in bovine [51] and human [34, 52]. Indeed, a negative relationship between global DNA breaks and SSB and developmental competency of fertilised embryos was found herein, suggesting that both global DNA damage and extensive SSB in sperm strongly compromise the embryo ability to develop after very early embryo stages. Importantly, not only were global DNA breaks and DSB found to negatively affect the percentages of early blastocysts/blastocysts and hatching/hatched blastocysts, but DSB was also seen to influence negatively the developmental potential of morulae. These results are in agreement with previous reports in humans, mice, cattle and goats, in which embryos produced with sperm containing DSB showed delays in their developmental kinetics and, ultimately, lower implantation rates and miscarriage within the first trimester [18–20, 53, 54]. Previous reports in mice proposed that extensive sperm DSB may probably exceed the oocyte repair capacity; consequently, paternal DNA replication may be delayed leading to embryonic developmental arrest [20]. Another hypothesis would be that sperm DSB could potentially lead to chromosome aberrations and mutations during early embryonic development, which could lead to cell death, thus inhibiting embryo development [53, 55, 56]. Indeed, the negative impact of DSB on morula developmental competency reported in the present work may be explained by the fact that it is not until the morula stage when chromosome aberrations trigger G1/S and G2/M checkpoints [57], which are likely to activate apoptotic mechanisms and avoid blastocyst formation [58]. Interestingly, as it has been already observed in human embryos [19], the present study also found that SSB do not seem to have an impact on embryo kinematics in porcine; however, further studies using time-lapse technologies are needed to confirm these observations. As previously hypothesised in humans, this could result from the capacity of zygotes to repair SSB since the complementary DNA strand is present [19]. Either way, the present study reinforces the idea that DSB have a dramatic, detrimental impact on mammalian embryo development and, for this reason, their assessment may contribute to increasing the efficiency of ART procedures.

The assessment of sperm DNA damage has been extensively proved to have a strong predicting ability for human fertility [27, 59]. Regarding the tests evaluating that damage, TUNEL, SCSA and Comet assays have been shown to be the most powerful [60, 61]. The use of more advanced methods, such as the Comet assay, however, is interesting due to: (i) its inherent ability to discriminate DSB and SSB [42], (ii) its high reproducibility and sensitivity [39, 62]; and (iii) its ability to equally detect breaks in protamine and histone-bound chromatin [39]. Considering this and the results presented herein, this work also evaluated whether including Comet assay to the conventional semen analysis could improve fertility prediction. Our data showed that, while the Comet assay was unable to predict fertilisation rate on day 2, including the evaluation of sperm DNA integrity through this technique to the traditional spermiogram had an additive effect, depicting an excellent discriminant value for predicting the percentage of blastocysts on day 6. This did not come as a surprise as we observed a relationship between the different types of SDF and embryo development parameters on day 6, but not between SDF and fertilisation rate on day 2. In addition, while this is the first report including the Comet assay to the routine semen analysis in livestock, previous studies in pigs [43, 50] and cattle [49, 63–66] already traced the clinical significance of other sperm DNA fragmentation assays. The present work, therefore, confirms using an animal model that routine testing of DNA integrity improves assisted reproduction outcomes, as previously advised for humans [27]. Also, the establishment of this relationship in porcine enables future studies assessing the effects of different putative treatments or genotoxic compounds on sperm DNA integrity, thus helping in the prevention and diagnosis of human reproductive disorders. In addition, future studies including the use of ICSI in animal models may help address whether sperm DNA fragmentation status differently affects IVF and ICSI outcomes.

## Conclusions

Sperm DNA damage has been previously found to influence fertility in mammalian species. Yet, no report has exhaustively evaluated the relationship of sperm SSB and DSB with sperm quality parameters, oocyte fertilisation and embryo development in porcine. The results of the present work concluded that SSB and DSB have a different impact on pig sperm quality parameters. Moreover, although sperm DNA damage does not seem to be related to the sperm ability to fertilise the oocyte, the present report evidences that while SSB are correlated to the amount of embryos observed on day 6, DSB compromise the percentage of embryos reaching the blastocyst stage. Importantly, our data support that the combination of the two Comet variants with conventional sperm quality parameters achieves very high discriminant value for embryo development outcomes. For all these reasons, this work sets a useful model to study how genotoxic agents inducing sperm DNA fragmentation affect fertility.

## Materials and methods

### Reagents

Unless stated otherwise, all reagents used in the present study were of analytical grade and purchased from Sigma (Merck, Darmstadt, Germany). Fluorochromes were acquired from ThermoFisher Scientific (Waltham, MA, USA).

### Animals and samples

All semen samples used in the present study were provided by a local farm (Gepork S.L.; Masies de Roda, Spain), which follows the ISO certification (ISO-9001:2008). All the procedures that involved animals were performed by the AI centre in accordance with the EU Directive 2010/63/EU for animal experiments, the Animal Welfare Law issued by the Regional Government of Catalonia, and the current regulation on Health and Biosafety issued by the Department of Agriculture, Livestock, Food and Fisheries, Generalitat de Catalunya, Spain. As no animal was manipulated to conduct the present experiment, since ejaculates were commercially acquired from a local farm (AI-centre), no permission from an Ethics Committee was required.

Ejaculates from healthy and sexually mature Pietrain boars (1–3 years old) were collected using the gloved-hand method. Immediately after collection, semen samples were diluted to a final concentration of 33 × 10^6^ sperm/mL using a commercial extender (Vitasem LD, Magapor S.L., Zaragoza, Spain) and stored at 17 °C for 24 h.

### Experimental design

Twenty-four ejaculates from 24 boars (one ejaculate per boar) were used to conduct the analyses described below. Each ejaculate, considered as a biological replicate, was split into three aliquots: the first was used to assess sperm quality, in terms of sperm motility, morphology and viability; the second was intended to IVF; and the third aliquot was stored at – 80 °C until alkaline and neutral Comet assays were carried out.

### Evaluation of sperm quality

#### Sperm motility

Sperm motility was assessed through a computer-assisted sperm analysis (CASA) system (Integrates Sperm Analysis System, ISAS V1.0; Proiser S.L.; Valencia, Spain) and Olympus BX41 microscope (Olympus; Tokyo, Japan) with a negative phase contrast field (Olympus 10 × 0.30 PLAN objective, Olympus). Semen samples were incubated for 15 min at 38 °C, and 5 µL of each sample were analysed in a pre-warmed Leja20 counting chamber (Leja Products BV; Nieuw-Vennep, The Netherlands). Two technical replicates were examined, evaluating 1000 sperm per replicate.

Several sperm velocity parameters were recorded: VSL, VAP, curvilinear velocity (VCL), amplitude of lateral head displacement (ALH), beat-cross frequency (BCF), LIN, percentage of straightness (STR) and WOB. Total motility and progressive motility were also recorded, and sperm were considered motile when VAP was ≥ 10 µm/s, and progressively motile when STR was over 45%.

#### Sperm morphology

After diluting semen samples with 0.12% formaldehyde in saline solution (PanReac AppliChem; Darmstadt, Germany; 1:1, v:v), sperm morphology was analysed under a phase-contrast microscope at 1000 × magnification (Nikon Labophot; Nikon; Tokio, Japan). Two hundred sperm cells were counted and those without morphology alterations were considered as normal. Moreover, primary and secondary alterations were recorded [67].

#### Sperm viability assessment

The LIVE/DEAD sperm viability kit (Molecular Probes, Eugene, OR, USA) following the protocol of Garner and Johnson [68] was used to evaluate plasma membrane integrity. This kit includes SYBR-14, which stains the nuclei of all sperm, and propidium iodide (PI), which only stains those of sperm that have lost their plasma membrane integrity. In brief, semen samples were diluted to a final concentration of 4 × 10^6^ sperm/mL in phosphate buffered saline 1 × (PBS). Next, sperm were stained with SYBR-14 (final concentration: 32 nM) and PI (final concentration: 7.5 µM) at 38 °C in the dark for 15 min. Next, stained samples were analysed using a CytoFLEX cytometer (Beckman Coulter; Fullerton, CA, USA). SYBR-14 fluorescence was detected by the fluorescein isothiocyanate (FITC) channel (525/40), and that of PI through the PC5.5 channel (690/50). Both fluorescent probes were excited with a 488-nm laser, and no spill compensation was applied. For each sample, three technical replicates containing at least 10,000 sperm were evaluated. Throughout the entire experiment, flow rate, laser voltage and sperm concentration remained unchanged. The percentages of viable (SYBR-14^+^/PI^−^) and non-viable sperm (SYBR-14^−^/PI^+^ and SYBR-14^+^/PI^+^) were recorded and used for the subsequent statistical analysis.

### Oocyte maturation, in vitro fertilisation, and embryo culture

First, ovaries from pre-pubertal gilts were collected at a local abattoir (Frigorífics Costa Brava; Riudellots de la Selva, Girona) and transported to the laboratory in 0.9% NaCl supplemented with 70 µg/mL kanamycin at 38 °C. Cumulus-oocyte complexes (COC) were retrieved from follicles and only those with complete and compact cumulus mass were selected and washed in Dulbecco’s PBS (Gibco, ThermoFisher) supplemented with 4 mg/mL of BSA.

For oocyte maturation, TCM-199 (Gibco) supplemented with 0.57 mM cysteine, 0.1% (w:v) polyvinyl alcohol, 10 ng/mL human epidermal growth factor, 75 µg/mL of penicillin-G potassium, and 50 µg/mL of streptomycin sulphate was used. Groups of 40–50 COCs were transferred to a four-well multi-dish (Nunc, ThermoFisher; Waltham, MS, USA) containing 500 µL of pre-equilibrated maturation media supplemented with 10 IU/mL equine chorionic gonadotropin (eCG; Folligon; Intervet International B.V.; Boxmeer, The Netherlands) and 10 IU/mL human chorionic gonadotropin (hCG; Veterin Corion; Divasa Farmavic S.A.; Gurb, Barcelona, Spain). After 20–22 h, oocytes were transferred to 500 µL of pre-equilibrated maturation media without hormones.

For the fertilisation protocol, denuded mature oocytes were placed in 50-µL drops of pre-equilibrated IVF medium containing 1 mM caffeine. The basic medium used for IVF was a modified Tris-buffered medium [69]. After adjusting semen samples to a final concentration of 1000 sperm per oocyte in IVF medium, oocytes and sperm were co-incubated for 5 h.

The presumptive zygotes were washed and transferred (40 zygotes/well) into a four-well multi-dish containing 500 μL of NCSU23 medium [70] supplemented with 0.4% BSA, 0.3 mM pyruvate and 4.5 mM lactate. After 2 days, cleaved embryos were counted to calculate the fertilisation rate; embryos were changed to NCSU23 medium supplemented with 0.4% BSA and 5.5 mM glucose, and cultured for 5 days. Embryos were classified following Balaban and Gardner [71] criteria and the percentages of morulae, early blastocysts/blastocyst, hatching/hatched blastocysts and total embryos (sum of morulae, early blastocysts/blastocyst and hatching/hatched blastocysts) were calculated on day 6 post-fertilisation. Moreover, two different ratios were determined: (i) the developmental potential of morulae on day 6, calculated as the percentage of early blastocysts/blastocysts plus hatched/hatching blastocysts divided by the percentage of morulae; and (ii) the developmental competency of fertilised embryos, calculated as the ratio between the number of embryos on day 2 and on day 6.

All procedures (oocyte maturation, IVF, and embryo culture) were carried out at 38.5 °C under a humidified atmosphere of 5% CO_2_ in air. Each of the 24 ejaculates was used as a biological replicate, obtaining at least 40 zygotes per semen sample.

### Neutral and alkaline Comet assays

The neutral Comet assay was used to quantify the amount of DSB, and the alkaline Comet assay was conducted to determine the whole amount of DNA breaks, including both SSB and DSB. In order to infer the amount of SSB, the neutral Comet OTM was subtracted from the alkaline Comet outcome. The protocols used for both Comet assays were previously adapted to pig sperm by Ribas-Maynou et al. [42].

#### Sperm fixation and lysis

First, samples were diluted to 5 × 10^5^ sperm/mL, and mixed with low melting point agarose (37 °C) at a final concentration of 0.66%. Quickly, two drops of the mixture (6.5 µL each) were poured onto two agarose pre-treated slides, one designated for neutral Comet and the other for alkaline Comet, and covered with an 8-mm round coverslip. Thereafter, agarose was allowed to jellify at 4 °C for 5 min and coverslips were gently removed. Both slides were incubated in three lysis solutions: (1) 0.8 M Tris–HCl, 0.8 M DTT and 1% SDS for 30 min; (2) 0.8 M Tris–HCl, 0.8 M DTT and 1% SDS for 30 min; and (3) 0.4 M Tris–HCl, 0.4 M DTT, 50 mM EDTA, 2 M NaCl, 1% Tween20 and 100 µg/mL Proteinase K for 180 min.

#### Electrophoresis

Electrophoresis was differently conducted depending on the Comet variant. For neutral Comet, slides were electrophoresed in TBE buffer (0.445 M Tris–HCl, 0.445 M boric acid and 0.01 M EDTA; pH = 8) at 1 V/cm for 4 min, and then washed in 0.9% NaCl for 2 min. For alkaline Comet, slides were denatured in cold (4 °C) alkaline solution (0.03 M NaOH, 1 M NaCl) for 5 min, and electrophoresed in an alkaline buffer (0.03 M NaOH, pH = 13) at 1 V/cm for 4 min.

#### Neutralization, dehydration, and staining

Both electrophoresed slides were incubated in neutralization solution (0.4 M Tris–HCl, pH = 7.5) for 5 min, dehydrated in ethanol series (70%, 90% and 100%) for 2 min each, and allowed to dry in horizontal position. Staining was conducted using 5 µL of 1 × Safeview DNA stain (NBS biological, Huntingdon, UK), and covered with a 20 × 20 coverslip.

#### Imaging and analysis

An epifluorescence microscope (Zeiss Imager Z1, Carl Zeiss AG, Oberkochen, Germany) was used to observe Comets. Captures of at least 100 sperm cells per sample were conducted at 100 × magnification and resolution of 1388 × 1040 pixels, through Axiovision 4.6 software (Carl Zeiss AG, Oberkochen, Germany). Exposure time was adjusted in each capture to avoid overexposure of staining.

The quantitative analysis of the fluorescence intensity of Comet heads and tails was conducted through the open-access CometScore v2.0 software (Rexhoover, www.rexhoover.com). After automatic analysis, a manual review of each analysed Comet was conducted to remove captures not corresponding to cells, overlapping comets, or those that showed impurities that affected head or tail signal. Also, this review served to correct any inaccurate interpretation of Comet heads by the software. At this point, if the final Comet number was less than 100, more captures were performed until this figure was reached.

For the quantification of the amount of DNA breaks, OTM calculated as (Tail mean intensity − Head mean intensity) × Tail DNA/100, was chosen as a reference parameter [72].

**Fig. 3:**
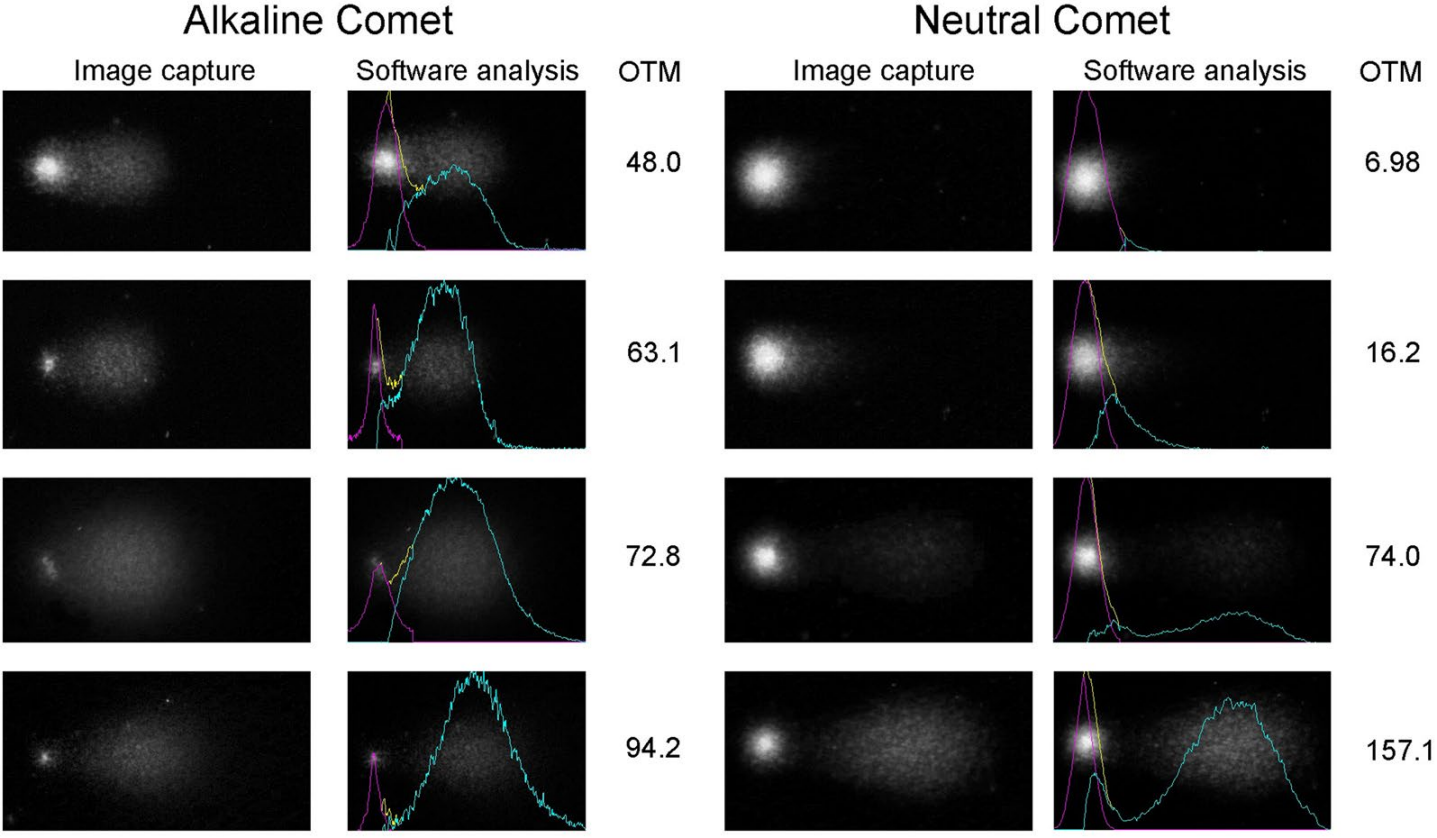
Representative images for alkaline and neutral Comet assay, and their respective analysis using the Cometscore v2 software. Purple lines indicate the intensity of the comet core, blue lines indicate the intensity of the comet tail, and yellow lines indicate the superposition between core and tail. *OTM* olive tail moment

A representative composition of images for the alkaline and neutral Comet assays, including the analysis of DNA damage conducted by the CometScore v2.0 software is shown in Fig. 3.

### Statistical analysis

Data were analysed through GraphPad Prism 8.0 Software (GraphPad, San Diego, USA), and Statistics Package for Social Sciences (SPSS) ver. 25.0 (IBM Corp.; Armonk, NY, USA). For all tests, the level of significance was set as *P* ≤ 0.05. First, normal distribution and homogeneity of variances were determined with Shapiro–Wilk and Levene tests, respectively. Thereafter, Spearman correlations between sperm DNA damage and sperm quality and IVF outcomes were run, and associations were assessed through multiple linear regression tests.

Subsequently, to determine the discriminant relevance of each DNA damage and sperm quality parameter for fertilisation on day 2 and total blastocyst percentage on day 6, these two IVF outcomes were divided into two groups below and above the median. A ROC analysis was used to determine the AUC of each variable, and the discriminant relevance was graded as: 0.0–0.5 no discriminant value, 0.5–0.6 failed discriminant value, 0.6–0.7 poor discriminant value, 0.7–0.8 fair discriminant value, 0.8–0.9 good discriminant value, and 0.9–1 excellent discriminant value. For all DNA damage and sperm quality parameters, sensitivity, specificity, and odds ratio were recorded.

Finally, in order to address if the addition of sperm DNA damage to the conventional semen analysis could have a higher discriminant value, a Principal Component Analysis (PCA) was generated including neutral OTM, alkaline OTM—neutral OTM, progressive motility, total motility, kinematic parameters, morphology and viability. These parameters were sorted into one PCA component, and the obtained data matrix was rotated through the Varimax procedure with Kaiser normalisation. Variables with a loading factor higher than 0.6 and lower than 0.3 in the rotated matrix were selected. The resulting coefficients were used to calculate regression scores that were assigned to each spermatozoon, and the variable was used to calculate a ROC curve for the prediction of fertilisation and blastocyst rates.

## Abbreviations

ALH: Amplitude of lateral head displacement
ART: Assisted reproductive techniques
AUC: Area under the curve
BCF: Beat-cross frequency
CASA: Computer-assisted sperm analysis
COC: Cumulus-oocyte complexes
DSB: Double-strand DNA breaks
eCG: Equine chorionic gonadotropin
FITC: Fluorescein isothiocyanate
hCG: Human chorionic gonadotropin
ICSI: Intracytoplasmic sperm injection
IVF: In vitro fertilisation
LIN: Percentage of linearity
OTM: Olive tail moment
PCA: Principal component analysis
PI: Propidium iodide
ROC: Receiver operating characteristic
ROS: Reactive oxygen species
SCD: Sperm chromatin dispersion
SCSA: Sperm chromatin structure assay
SDF: Sperm DNA fragmentation
SSB: Single-strand DNA breaks
STR: Percentage of straightness
TUNEL: Terminal deoxynucleotidyl transferase dUTP nick end labelling
VAP: Average path velocity
VCL: Curvilinear velocity
VSL: Straight-line velocity
WOB: Motility parameter wobble
8OHdG: 8-hydroxy-2′deoxyguanosine

## Declarations

### Availability of data and materials

The datasets used and/or analysed during the current study are available from the corresponding author on reasonable request.
